# Rapid phenotyping of crop root systems in undisturbed field soils using X-ray computed tomography

**DOI:** 10.1186/s13007-015-0084-4

**Published:** 2015-08-28

**Authors:** Johannes Pfeifer, Norbert Kirchgessner, Tino Colombi, Achim Walter

**Affiliations:** Institute of Agricultural Sciences, Swiss Federal Institute of Technology in Zurich (ETH Zürich), Universitätstrasse 2, 8092 Zurich, Switzerland

**Keywords:** Non-destructive root phenotyping, X-ray computed tomography (CT), High throughput, Image analysis, Field soil, Root growth dynamics, Root system architecture (RSA), Root thickness

## Abstract

**Background:**

X-ray computed tomography (CT) has become a powerful tool for root phenotyping. Compared to rather classical, destructive methods, CT encompasses various advantages. In pot experiments the growth and development of the same individual root can be followed over time and in addition the unaltered configuration of the 3D root system architecture (RSA) interacting with a real field soil matrix can be studied. Yet, the throughput, which is essential for a more widespread application of CT for basic research or breeding programs, suffers from the bottleneck of rapid and standardized segmentation methods to extract root structures. Using available methods, root segmentation is done to a large extent manually, as it requires a lot of interactive parameter optimization and interpretation and therefore needs a lot of time.

**Results:**

Based on commercially available software, this paper presents a protocol that is faster, more standardized and more versatile compared to existing segmentation methods, particularly if used to analyse field samples collected in situ. To the knowledge of the authors this is the first study approaching to develop a comprehensive segmentation method suitable for comparatively large columns sampled in situ which contain complex, not necessarily connected root systems from multiple plants grown in undisturbed field soil. Root systems from several crops were sampled in situ and CT-volumes determined with the presented method were compared to root dry matter of washed root samples. A highly significant (*P* < 0.01) and strong correlation (R^2^ = 0.84) was found, demonstrating the value of the presented method in the context of field research. Subsequent to segmentation, a method for the measurement of root thickness distribution has been used. Root thickness is a central RSA trait for various physiological research questions such as root growth in compacted soil or under oxygen deficient soil conditions, but hardly assessable in high throughput until today, due to a lack of available protocols.

**Conclusions:**

Application of the presented protocol helps to overcome the segmentation bottleneck and can be considered a step forward to high throughput root phenotyping facilitating appropriate sample sizes desired by science and breeding.

**Electronic supplementary material:**

The online version of this article (doi:10.1186/s13007-015-0084-4) contains supplementary material, which is available to authorized users.

## Background

Increasing the throughput for quantitative characterization of plant root system architecture (RSA) is important for plant breeding [1] and to come to an improved understanding of root–soil interactions [2–4]. In the context of root phenotyping, X-ray computed tomography (CT) has become a powerful tool [5]. Compared to rather classical, destructive methods, in which roots are first washed out of the soil, imaged and then analyzed with commercially available (e.g. WinRHIZO, Regent Instruments Inc., Sainte-Foy, Québec, Canada) or custom-made software (e.g. so-called ‘shovelomics’ approaches as described in [6, 7]), CT encompasses various advantages. In particular, the possibility to follow the same individual root growing over time and to study dynamic root growth and development processes in pot experiments, and, in addition, the opportunity to explore the unaltered configuration of the 3D RSA interacting with a real field soil matrix, makes CT a unique tool for plant research. Moreover, when using destructive methods, in which roots are washed out of the soil, fine roots frequently break off and are washed away, while they can be analyzed in CT scans if the spatial resolution is appropriate [8].

Yet, using CT, the throughput suffers from the bottleneck of rapid and standardized segmentation methods to extract root structures [4, 9]. Indeed, unaltered RSA can be analyzed by CT, but the segmentation of the root (optical separation of root and soil) is done to a large extent manually and therefore requires a lot of time. Segmentation is usually performed by defining a local threshold for gray values of the CT voxels, classifying them either as root or non-root. These local thresholds often vary throughout a sample due to heterogeneities in the substrate and CT-artefacts. The complete reconstruction of a root system therefore requires a lot of interactive parameter optimization and interpretation, which is supported by software tools that allow identification of connected root systems (e.g. by region growing algorithms as used in [10–12]). Yet, the premise of a completely connected root system can also lead to difficulties. Algorithms that follow root voxels in CT image stacks slice by slice [13, 14] can be confused, if a root seems to be interrupted due to surrounding soil heterogeneities or if roots are only a few voxels in diameter or if multiple root systems contained in soil cores from a field experiment are investigated. These root systems typically consist of cut segments of roots from multiple plants contained in the cylinder of a soil core. Besides this difficulty of segmenting unconnected roots, root segmentation is far more difficult in undisturbed field soils compared to sieved soil filled in pots due to further obstacles. Undisturbed soils frequently contain much higher amounts of organic particles, which are commonly removed in pot experiments by means of sieving the soil. Those organic particles typically have gray values similar to those of roots. Moreover, undisturbed soil samples often show more inhomogeneous moisture distribution compared to sieved and homogenized soils.

Since an ultimate goal of root phenotyping is the characterization of realistic root systems of plant stands in the field [15], it was the aim of the here presented approach to elaborate a reliable and fast protocol to segment root structures for single or multiple plants, which depends only minimally on the operator and which is applicable for simple root systems, but also for large, arbitrarily complex and unconnected root systems.

## Results and discussion

**Fig. 1:**
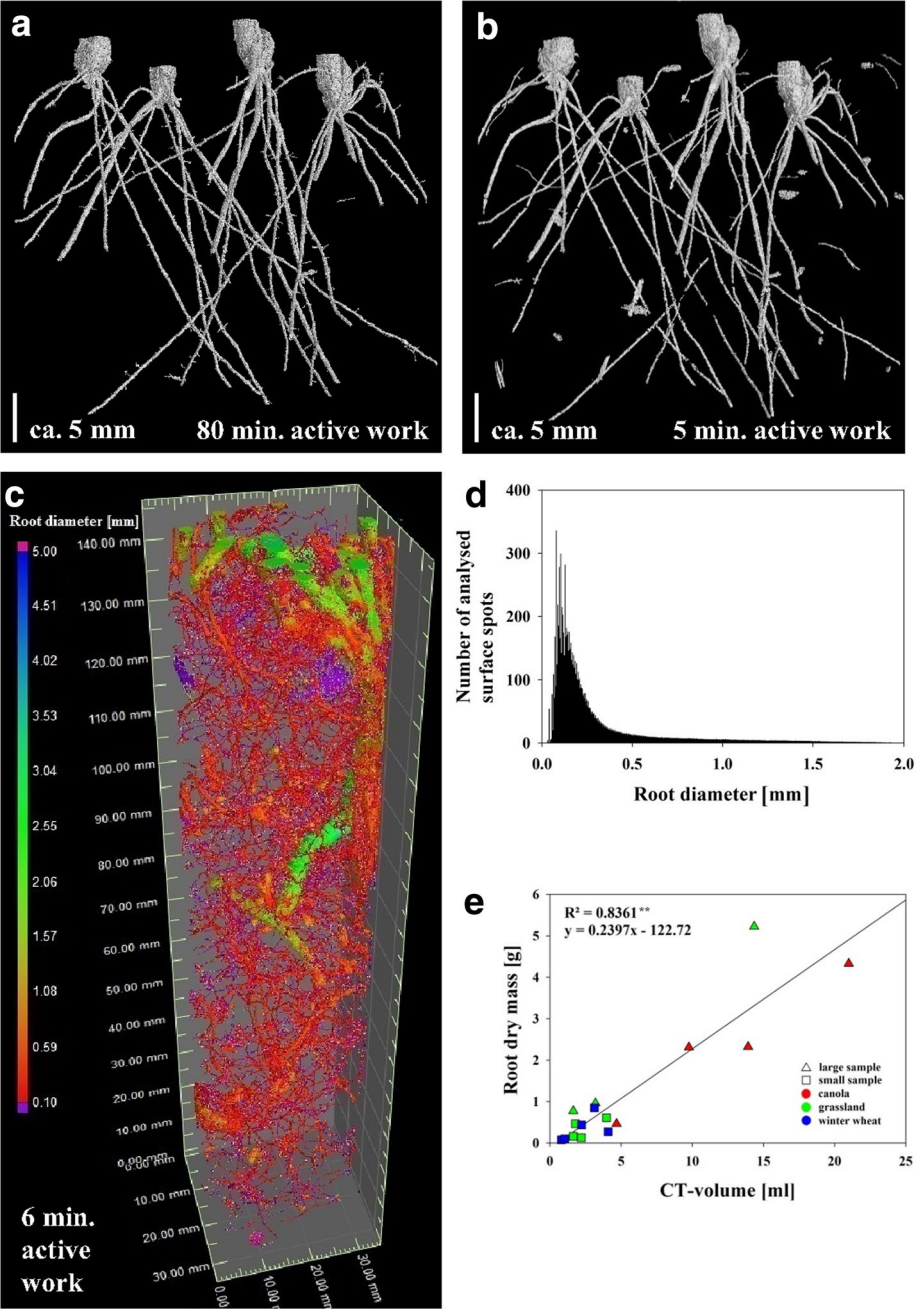
CT results. 3D RSAs of barley seedlings (**a**, ** b**) grown for 14 days in a 6 cm diameter pot filled with Haplic luvisol (Campus Klein-Altendorf) and grass–legume mixture (**c**) grown for 24 months in an Pseudogleyed Cambisol (Agroscope research station Reckenholz), sampled in undisturbed fashion in situ using a 3.4 cm diameter cylinder. In **a** roots were segmented by a region-growing algorithm (80 min active working time), and in **b**, **c** the roots were segmented using the presented protocol (5 min active working time). Root diameter distribution of **c** is shown in color-coded scale (+1 min of active working time for diameter measurement = 6 min). The distribution of the frequency of root surface elements with a specific root diameter was calculated (**d**). Root dry mass density is plotted against root volume as reconstructed by CT (**e**); **significant, linear correlation on *P*-level 0.01 (*n* = 18)

Based on new software, this paper presents a protocol that is faster, more standardized and more versatile compared to existing methods, particularly if used to analyse field samples. This opens the door to more automated, high-throughput root phenotyping. All image processing steps of the presented protocol took about 5 min of active working time per sample and 30 min of computation time including data loading, segmentation and size filtering. In another recently published method [9], in which a segmentation based on gray values was performed, a minimum of 1 h of active segmentation time per sample has been specified. In contrast to most other approaches, this method allowed for extracting unconnected roots systems, similar to the here presented approach. In a study applying a region growing algorithm [10], the authors mention a required timeframe of 50 min before the actual manual segmentation was performed (the actual segmentation time was not mentioned; in our work an additional 80 min was needed for the actual segmentation with the region growing algorithm, which is shown in Fig. 1a). Using RooTrak software [13], the authors report 15–60 min to process the CT data. Moreover, they could not extract unconnected root systems.

Analyzing RSA from field soil can be done with conventional destructive methods too, which are regularly very time-consuming. Indeed, applying so-called ‘shovelomics’ approaches for example, in which RSA traits of maize plants can be visually scored after destructive excavation and washing out from soil, 5–10 min of active working time per sample are needed [6, 7]. Though, shovelomics are not suitable for delicate root systems from field-grown plants (e.g. of cereals), as roots of these plants agglomerate after washing. For delicate root systems of field-grown plants several fundamentally different methods are available, which are all very laborious, as for example WinRHIZO [9, 16] and the profile wall method, originally described by [17]. For application of WinRHIZO, the roots have to be washed even more thoroughly than for shovelomics, imaged and analyzed by the software, which frequently needs, depending on the soil volume to be analyzed, significantly more than 1 h of active working time per sample. Using the profile wall method, several tedious steps have to be performed including the excavation of a walk-in cored hole in the field, preparing the wall in several steps, and counting roots manually using a counting frame. Depending on the number of plants analyzed at the profile wall, several hours or days per profile wall are frequently needed to perform the data acquisition.

The root systems extracted in the framework of the presented segmentation protocol (Fig. 1b, c) showed a high level of complexity and integrity. Consequently, various RSA parameters, such as the number of lateral roots, branching angle or root diameter (Fig. 1c, d) that are interesting for both research and breeding, can be analyzed by the same software used in this work [16]. A highly significant (*P* < 0.01) and strong correlation (R^2^ = 0.836) was found for root dry matter of washed root samples and root volumes determined for the same roots with the presented method (Fig. 1e). Given that the CT volume is closely correlated to the fresh weight of the root, the slope of the regression line indicates an average dry weight content of 24 %. This value appears plausible as the samples contained many storage roots, e.g. from *Brassica napus* and *Medicago sativa* and storage roots frequently show root dry weight contents between 25 and 35 % [18]. It can be expected that not all roots are identified, both using CT and root washing. However, the correlation shows that the error of missed roots is similar, making the results of both methods comparable.

Previous work has demonstrated the applicability of X-ray computed tomography to investigate effects of various environmental conditions on RSA traits and to study relevant root–soil-interactions, such as effects of soil physical properties on rhizosphere functions [16, 19, 20]. However, in those studies typically disturbed field soil was used, which was sieved to remove organic residues and filled into rather small pots. As plants were sown directly in those pots, the scans usually contained whole root systems. Moreover, the roots were typically resolved in comparatively high resolution due to commonly rather small pot diameters. All these provisions significantly relieve root segmentation. To the knowledge of the authors this is the first study approaching to develop a comprehensive segmentation method suitable for comparatively large columns sampled in situ which contain complex, not necessarily connected root systems from multiple plants grown in undisturbed field soil.

Studies on undisturbed soil samples are needed to increase the general understanding of root growth dynamics, root–soil interactions, root functions and their economic and ecological importance in agro-ecosystems. Available methods for this field of research are still underdeveloped. For instance, virtually all available methods for studying RSA traits of field samples result in a strong underestimation of the proportion of fine roots [8]. Fine roots, defined as roots thinner than 0.2 or 0.5 mm diameter [8], can make up more than 80 % of the root length of cereals [for a review see 8]. Using the presented protocol, it was observed for the grass–legume mixture samples, scanned at a resolution of 44 µm voxel size, that the major part of the root surface was formed by fine roots thinner than 0.25 mm diameter (Fig. 1c, d; maximum of histogram around 150 µm diameter). Similar observations were made for soil cores taken from wheat plots (data not shown). In future experiments, soil cores with even smaller diameter will be taken at selected positions. In these cores, the smaller voxel dimensions should allow segmentation of even thinner fine roots. Moreover, the use of advanced filtering algorithms, which make advantage of geometrical properties such as width-length or volume-surface ratios to eliminate remaining noise [9] should be possible in the near future. Those filters may help preserving small and unconnected root segments and could improve the accuracy of the extraction of the root systems, including fine root structures, particularly if other field soils containing higher amounts of organic matter are used. The intended application of refined filtering approaches will then facilitate extraction of further traits of RSA valuable for research and breeding.

## Conclusions

Application of the commercially available software VG Studio MAX 2.2 and the two applied add-on modules (‘Coordinate measurement’ and ‘Wall thickness analysis’) in combination with the presented protocol allows for time saving segmentation of arbitrarily complex and unconnected root systems, which can originate from pot experiments or can be collected in situ in the field. For this reason this protocol helps to overcome the segmentation bottleneck and can be considered a step forward to high throughput root phenotyping facilitating appropriate sample sizes desired by science and breeding. Moreover, a fast and simple way for a quantitative determination of root thickness distribution, which is an important but normally only very tediously determinable phenotypic trait, has been applied thereby. Further RSA parameters interesting for both research and breeding can be analyzed by the same software used here. Therefore, the application of the specified software in combination with the described protocol will result in a significant progress for a large spectrum of future studies performed in the field of crop phenotyping.

## Methods

### Plant material and collection of undisturbed soil samples

In order to demonstrate the potential of the method, its applicability to segment roots from four different crop systems [barley (*Hordeum vulgare* L. cv. Ascona), wheat (*Triticum aestivum* L. cv. CH Claro), canola (*B. napus* L. cv. Visby), and a grass–legume mixture (containing *M. sativa* L.)] in three different field soils (Haplic Luvisol from Campus Klein-Altendorf, Germany; Eutric Cambisol from ETH research station Eschikon, Switzerland; Pseudogleyed Cambisol from Agroscope Zürich-Reckenholz, Switzerland) was tested (Table 1). The segmented root volumes were then correlated with root dry matter of washed out roots (Fig. 1e). With the exception of barley, which was grown in a pot experiment (data from [11]), all plant samples (*n* = 18) were grown in situ for 8–24 months in the field and sampled in an undisturbed fashion (Table 1). Small samples with a diameter and height of 3.4 and 15 cm, respectively, were taken from winter wheat (*n* = 5) and from an intensively managed grass–legume mixture containing *M. sativa* L. (*n* = 4). Large samples of 10 cm diameter and height were excavated from the same grass–legume stand (*n* = 5) and from canola (*n* = 4). For collecting undisturbed samples, a custom-made stainless steel ring, sharpened only at the outside of the cutting edge to avoid compaction of the sample, was fitted to PVC cylinders (see measures in Table 1). Using a custom-made steel lid with holes to let the air escape, the cylinders were closed on top and gently pushed into the soil straight downwards with a hammer until the cylinders were just completely inserted in the soil. Next, samples were dug out using a spade. Protruding soil was removed with a knife. Then the samples were wrapped in aluminum foil to prevent soil drying. CT-scans were either performed at the same day or samples were stored at 4 °C for maximum 1 day until CT-scans were performed. After scanning the roots were thoroughly washed out of the soil using sieves with a mesh size of 1 mm. In order to minimize the loss of roots due to the washing procedure the soil was sieved 3 times. Afterwards the roots were dried for 3 days at 65 °C in a drying oven before the dry matter was determined.

**Table 1:** Soil and sample properties and scanning parameters for X-ray computed tomography

	Barley	Wheat	Canola	Grass–legume mixture
*Cultivation parameters*				
Experimental conditions	Pot experiment (controlled conditions)	Samples collected in situ in the field	Samples collected in situ in the field	Samples collected in situ in the field
Soil type	Klein-Altendorf: Haplic Luvisol, sieved	Eschikon: Eutric Cambisol; Reckenholz: Pseudogleyed Cambisol	Eschikon: Eutric Cambisol	Reckenholz: Pseudogleyed Cambisol
Plant age (months)	0.5	8	8	24
Cylinder internal diameter (cm)	6	3.4	10	3.4 (small samples) and 10 (large samples)
Cylinder material	PE	PVC	PVC	PVC
Number of plants per cylinder	4	1	1	>1
*Acquisition parameters*				
Height of scanned part of root system (cm)	16	15	10	15 (small samples) and 10 (large samples)
Height of analyzed part of root system (cm)	4	15	10	15 (small samples) and 10 (large samples)
Voxel size (mm)	0.05	0.044	0.120	0.120 (large samples) and 0.044 (small samples)
Binning	1 × 1	2 × 2	2 × 2	2 × 2
Current (µA)	450	350	450	450 (large samples) and 350 (small samples)
Voltage (kV)	180	120	120	120
Number of images per subscan	3000	1600	1600	1600
Averaged images	3	1	1	1
Skipped images	1	0	0	0
Filtering	0.1 mm copper	0.1 mm copper	0.4 mm copper	0.4 (large samples) and 0.1 mm (small samples) copper
Observation ROI option	yes	yes	yes	yes
Exposure time per image (ms)	200	131	1000	131 (small samples) and 1000 (large samples)
Scan duration (min)	120	41	30	30
Multiscan and number of subscans	Yes (3)	Yes (3)	No	Small samples: yes (3), large samples: no
*Reconstruction parameters*				
Downscaling to unsigned 16 bit	Yes	Yes	Yes	Yes
Reference ROI	Yes	Yes	Yes	Yes
Auto scan optimizer	Yes	Yes	Yes	Yes
Beam hardening correction	Assuming different materials, value 4	Assuming different materials, value 3.6	Assuming different materials, value 3.6	Assuming different materials, value 3.6

### CT-measurement

X-ray computed tomography scans were performed at the Swiss Federal Institute of Technology Zurich (ETH Zürich, Switzerland) using a phoenix v|tome|x s 240 X-ray scanner (GE Sensing & Inspection Technologies GmbH, Wunstorf, Germany). Two different configurations of acquisition parameters for tomography were chosen for the two different sample sizes (Table 1). Volumes were reconstructed using the software datos|x (GE Sensing & Inspection Technologies GmbH, Wunstorf, Germany). For reconstruction (in 32-bit float) an auto-scanoptimization and a beam hardening correction were performed. In case of multiscans, the single subscans were combined while the data were reconstructed. For this reason, data from multiscans could be analyzed together. Very slight gray value differences could be observed in the transitions from one subscan to the other. However, for the subsequent analysis it was not necessary to normalize for those differences in order to achieve seamless transitions.

### Data analysis

**Fig. 2:**
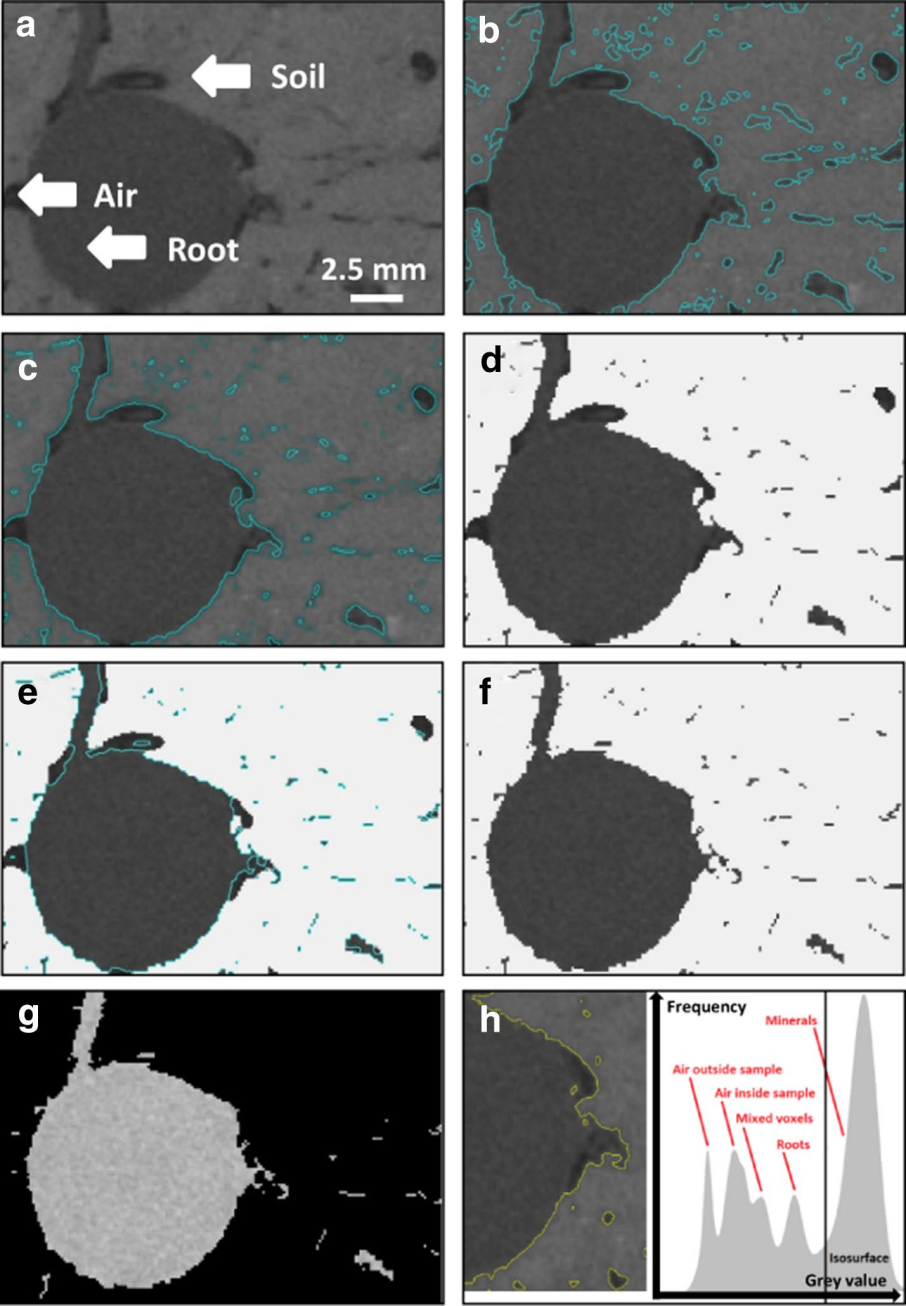
Steps of the segmentation protocol. Original X-ray CT volume of grass-legume mixture sample (details given in Table 1) showing roots, air-filled pores and soil (**a**). First step: advanced surface determination of the soil. The surface is shown as a *blue line* around the soil aggregates (**b**). Second step: dilatation of the region of interest (ROI), here 1 voxel, to add mixed voxels. The contour of the dilated surface is shown as a bright *blue line* (**c**). Step three: subtraction of the dilated ROI from a ROI containing the whole volume. Only roots and pores remain in the resulting volume (**d**). Step four: detection of the root surface (shown as a *blue line*) (**e**). Step five: the volume containing the roots and remaining noise (**f**) is exported to MatLab and filtered therein. The resulting, filtered volume containing only roots is shown in (**g**). The peaks of the gray values of air, mixed voxels, roots and minerals shown in the histogram are not completely separated (**h**)

Volume data analysis was performed by VG Studio MAX 2.2 software (Volume Graphics GmbH, Heidelberg, Germany) and the add-on modules ‘Coordinate measurement’ (Advanced surface determination) and ‘Wall thickness analysis’. Original images (32-bit float) were downscaled to 16-bit unsigned integer. In general, pores and air are of lower gray values than roots, which are of lower gray values than soil components. Unfortunately, the four mentioned objects are not easily separable by simple global thresholding [9, 10]. In the first step of this protocol, all mineral structures (soil) were segmented in the original reconstructed volume (Fig. 2a) using the ‘Advanced surface determination’-tool by manual selection of air as background and mineral parts as material using the ‘Define material by example area’-function (Fig. 2b). Applying the ‘Advanced surface determination’-tool, gray value thresholds can be continuously adjusted according to a preview window showing the resulting surface determination. The ‘Advanced surface determination’-tool refines the surface locally at several thousand locations along the object surface (here: soil aggregates) by a local adjustment according to the gradient of the gray values. The same gray value is reinterpreted according to the gray value of the neighboring voxels. This allows for a very precise determination of the surface to the target structure. A new region of interest (ROI) was generated from the surface, and attention was paid that preferably no root voxels but, where possible, all mineral voxels were included. In the second step (Fig. 2c), the ROI was dilated by 0.5–1 voxels in order to add mixed voxels at the border of the soil aggregates to air-filled pore spaces (also missing mineral voxels are commonly added by dilatation). Mixed voxels are formed due to volume averaging effects and frequently have gray values similar to root voxels, which would hinder root segmentation significantly [10]. In step three (Fig. 2d), the ROI containing mineral structures and mixed voxels was then subtracted from a ROI containing the whole volume so that only roots and pores filled with air and water remain in the resulting volume (Fig. 2d). Here, it proved very helpful to choose pots made of PVC as the gray value of PVC is very different to the one of roots and normally similar to the gray value of the mineral fraction. For this reason, the pot wall is included in the ROI containing mineral structures. In step four (Fig. 2e), the roots were segmented within the volume containing only roots and pore spaces analogously by manual selection of air as background and roots as material using the ‘Define material by example area’-function. A volume containing all roots and some remaining noise can be generated by extracting a new volume from the root surface. The fifth step comprises noise elimination, which was performed on exported tiff image stacks (Fig. 2f) by MatLab 8.0 (The Mathworks, Natick, Massachusetts, United States) using size thresholds. Using the MatLab algorithm (script provided as an.exe-file in Additional file 1, working on 16-bit unsigned integer) all structures smaller than 10,000 connected voxels were deleted. The denoised volumes were saved as tiff image stacks and imported to VG Studio MAX 2.2 for subsequent analysis (Fig. 2g).

Frequency distributions of root diameters were determined using the add-on module ‘Wall thickness analysis’ of the VG Studio MAX 2.2 software. Similar to the advanced surface determination algorithm the diameter of the root was calculated along the root surface (Fig. 1c, d). The surface determined by the ‘Advanced surface determination’-tool served as the starting contour. Several parameters and tolerance values can be manually adjusted using the ‘Wall thickness analysis’-tool, such as minimum and maximum thickness of the target structure and operating search angles.

In order to compare the presented protocol with another common method to extract root systems from CT volumes [10–12] the region growing tool from VG Studio MAX 2.2 was chosen (Fig. 1a). The tool is based on a region growing algorithm starting at manually selected seed points (here root material). As the local thresholds for segmenting roots commonly vary throughout the sample (due to heterogeneities in the substrate and CT-artefacts), it was, even though the adaptive mode was used, not possible to apply the algorithm to the entire CT volume. Instead, the search area of the algorithm needed to be restricted in 3D, which made it necessary to apply the algorithm in a plenty of single steps (by determining new seed points). In each step the newly segmented root structure was added to the already segmented part of the root system (saved as a ROI), and in each step the tolerance value needed to be adjusted manually to the local threshold.

## Additional file

Additional file 1: MatLab 8.0 script for noise elimination, provided as an .exe-file working on 16-bit unsigned integer.
